# Medical literature review: Search or perish

**DOI:** 10.12669/pjms.292.3418

**Published:** 2013-04

**Authors:** Younis Skaik

**Affiliations:** Younis Skaik, Lecturer and Researcher in Immunohaematology, Department of Laboratory Medicine, Faculty of Applied Medical Sciences, AL-Azhar University-Gaza, Palestine.

**Keywords:** Literature search

## Abstract

Literature review is a cascading process of searching, reading, analyzing, and summing up of the materials about a specific topic. However, searching the literature is like searching “a needle in a haystack”, and hence has been called “Cinderella”.^1^ Therefore, skills and effective pathways of searching the literature are needed to achieve high sensitive and specific results.

***Importance of searching the literature:***

A good review of previously published work has many advantages. Firstly, it prevents wasteful salami work, and hence saves financial resources. Secondly, it is a pathway of creating new idea from others’ work by filling the research gaps in the previous work and trying to know what is unknown about a specific topic. Steward^1^ has summarized the criteria of a good literature review.

***Literature search engines: pros and cons:***

It is worth noting that the researchers should have a previous knowledge about the different available literature searches such as PubMed, MEDLINE, CINAHL, Google Scholar, and many others. The characteristics of the most literature searches have been previously mentioned^2^, and the comparison between the efficiency and effectiveness of the two most popular literature searches (the PubMed and Google Scholar) have been extensively studied. The question arises which literature search should the authors use to obtain an appropriate results? The answer is the knowledge that the authors must know about the pros and cons of each literature search and the complementary pathways that should be used to retrieve most of the related work about a specific topic. There is an ongoing debate about the accuracy, precision, sensitivity, and specificity of either using the PubMed or a search engine (e.g., Google Scholar) as a major tool of searching the literature.^3^^-^^7^ However, combining the simplicity, speed, and the accessibility of the “grey” literature using the Google Scholar^8^ with the strengths of the PubMed is highly recommended and will retrieve highly sensitive and specific results. The “Net Generation” prefers using the Google Scholar rather than the PubMed. However, authors must appreciate the limitations of every pathway they use and to combine different pathways is always better to get an optimal literature results. 

***Searching the literature: An example:***

As searching the literature depends on the clinical question, selection of search terms, framing the questions, or key words, authors should spend time to consider different terms to avoid missing any article, which could be disastrous. Searching terms are the “bait fish” which are used as a bait to capture and retrieve most if not all of the related work about a specific topic.

***Example of good terms formatting:***

To the best of my knowledge, only two studies have investigated the level of β_3_ integrin in the serum of healthy donors. To find the two papers in the “haystack” try the following luring terms, using both Google Scholar and PubMed.

1. Serum β3 *vs* serum beta3.
2. Plasma B3 *vs *serum B3.
3. Serum beta3 and healthy *vs* serum beta3 and disease.

The only term which will retrieve successfully the two papers is “serum beta3 and disease” using both Google Scholar and the PubMed, however, much faster using Google.

***Top tips of searching the literature:***

Galaxies of tips have been suggested and many are available with videos and illustrations on the internet. Goggling the term “tips for searching the literature” will retrieve about 7.050.000 results. However, a very recent article suggested only four steps,^9^ and another recommended 18 steps^10^ for an effective literature searching. Having a good plan and enough time for framing the appropriate questions, as mentioned above in the example, will facilitate retrieving of the most if not all related work about a specific topic. One should always prefer combining different search terms and using the strengths of the two pals search engines (PubMed and Google Scholar) to achieve a high sensitive and specific search results. Maximum patience is desired in this stage by the authors to trap all related work, and keep in mind “keep Googling and PubMeding” will keep you on a safe side. Some additional tips for searching the literature are illustrated in Fig.1.

***Most wanted articles: the process of weeding out:***

After searching and collecting the literature, the authors need to weed out what they have collected and critically evaluate the sensitivity and specificity of the collected materials. This process is a vital to weed out the most wanted and related material about a specific topic. In addition, one can rapidly scan the dimensions of the topic and can find further key words and phrases for the future search. The collected materials can contain peer-reviewed and non-peer-reviewed materials; therefore, care should be taken while analyzing the search results. Further suggestions and recommendations on evaluating the collected literature have been suggested in different studies.^11^^-^^13^

**Fig.1:**
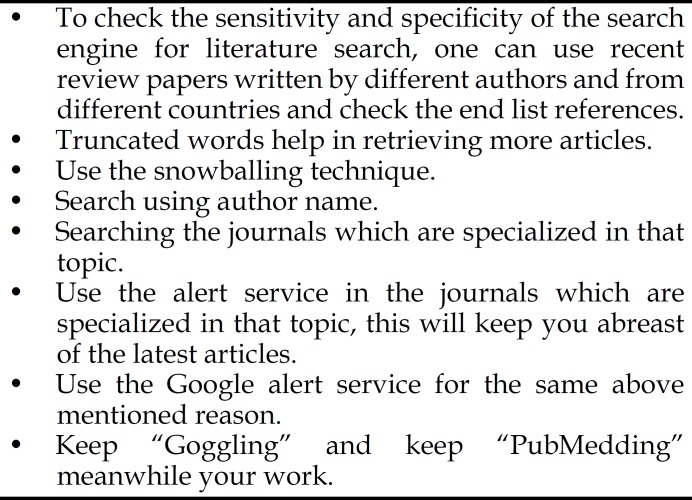
Hints for literature search.

***Acknowledge the chaotic or systematic search:***

The term “to the best of my knowledge” can be used to describe gently the probability of inadvertently missing of one article about a specific topic. However, missing an article may result either from a chaotic searching strategy, not all medical journals are indexed in the PubMed, inappropriate searching terms and key words, or missing the articles which are published in non-English languages. The junior authors and the “Net Generation” use this term more often in their writing, however, the senior reviewers will find it out very easily. Hence, it is always worthwhile to spend more time in framing and phrasing the sound terms, questions, and key words and to use these terms for capturing all related work about a specific topic and using the right search engines.

***Conclusions: Best search leads to best research:***

A searching plan is a perquisite step to get high accurate and precise results. The plan should detail all necessary key words and phrases that should be tried by the authors. Evaluation of the collected material is pivotal to assess the sensitivity and the specificity of both the search terms and the search engines used. Keeping in mind the literature review is one step in the chain of publishing the authors’ work, and best searching strategies lead to best research results and hence a worthy publications. 
